# Effects of food sources on food waste behaviour: evidence from urban regions of Vietnam

**DOI:** 10.1186/s40100-025-00445-8

**Published:** 2025-12-08

**Authors:** Minh Hai Ngo, Thanh Mai Ha, Bárbara Franco Lucas, Anh Duc Nguyen, Thi Lam Bui, Nhu Thinh Le, Mathilde Delley, Franziska Götze, Evelyn Markoni, Bao Duong Pham, Thomas A. Brunner

**Affiliations:** 1 Faculty of Economics and Management, Vietnam National University of Agriculture, Hanoi, Viet Nam; 2 Department of Economics, Swedish University of Agricultural Sciences, Uppsala, Sweden; 3 Food Science and Management, School of Agricultural, Forest and Food Sciences, Bern University of Applied Sciences, Burgdorf, Switzerland; 4 Department of Economics and Marketing, Fruit and Vegetable Research Institute, Hanoi, Viet Nam; 5 Bac Giang Agriculture and Forestry University, Bac Giang, Viet Nam; 6 Faculty of Accounting and Business Management, Vietnam National University of Agriculture, Hanoi, Vietnam

**Keywords:** Food sources, Food waste behaviour, Expired food, Experiment, Perceived food values

## Abstract

The relationship between food sources and food waste handling behaviour, though important in designing food waste reduction interventions, has been largely overlooked. In this context, this study investigates how food sources influence food waste behaviour. Specifically, it compares food waste management practices across food sources and examines the impact of food sources on consumers’ decisions to save food. We conducted an online survey in urban Vietnam and yielded 616 valid responses. The survey presented four scenarios capturing corresponding food sources for two hypothetical products: vegetables and pork, which were forgotten and expired, but still edible. They include (1) supermarket purchases, (2) food bought from a known farmer, (3) gifted party leftovers, and (4) gifted party leftovers sourced from a known farmer. The results show the lowest discard rate for the meat sourced from a known farmer (62%) and vegetables that are party leftovers (27%). Proportion test and bivariate probit regression confirm that food sourced from known farmers, which is either gifted leftovers or food bought by consumers, was more likely to be saved, as compared to that from supermarkets, suggesting higher perceived values of local food. The study suggests that fostering food waste reduction requires better consumer communication of food values associated with their perceived favourable food sources, especially local food.

## Introduction

Food waste, which refers to food discarded at retail and consumer levels, represents a prevalent global challenge with economic, social, and environmental aspects (FAO 2011). Roughly one-third of the total food produced is lost or wasted, amounting to around 1.5 billion tons annually at the cost of around 1 trillion USD. In addition, food waste and loss contribute roughly 8–10% of global greenhouse gas emissions, highlighting their significant environmental impact (UNEP 2024). Given that one in eleven people in the world is in chronic hunger in 2023 (FAO, IFAD, UNICEF, WFP, and WHO 2024), a drastic reduction in food waste can contribute to addressing the food insecurity problem. Since 60% of food waste is generated by households (UNEP 2024), food waste reduction at the household and individual levels will be an important driver of climate change mitigation and improved global food security.

Vietnam, an emerging country with a population of 106 million (GSO 2024), is also facing the food waste problem. Annually, nearly 9 million tons of consumable food are wasted in Vietnam, resulting in economic losses of approximately 4 billion USD, equivalent to about 2% of the national GDP (Ngan et al. 2024). Household food waste alone accounts for over 80% of all food discarded nationwide, around 7.3 million tons yearly (FoodCycle Science 2023). In urban regions, rapid economic development, the modernization of the food system, and the rise of the urban middle classes have changed food consumption practices (Ehlert 2016). Consequently, the amount of urban food waste per capita has increased significantly, particularly in large and fast-growing cities (Pham et al. 2021). A study in Da Nang city found that 87% of respondents reported wasting food at least two times a week (Pham et al. 2021). Another study from Hanoi estimates that the amount of food waste is 285 g/day/person in urban areas, which is as high as that in many developed cities and countries (Liu and Nguyen 2020). Tackling the food waste problem in urban regions of Vietnam is, therefore, urgently needed. To do so, an understanding of decision-making towards food wastage is crucial.

A growing body of research has shed light on the drivers behind individuals’ and households’ food waste (Cheng et al. 2025). Aydin and Yildirim (2021) found that consumers who view improper food disposal negatively and purchase only a sufficient quantity for consumption tend to generate less waste, suggesting the role of moral attitudes and shopping practices in food waste behaviour. Beliefs such as feeling guilty lead to food waste reduction, while food-related practices like eating out frequently and buying the best offers increase food waste volume (Delley and Brunner 2017; Mattar et al. 2018). In general, factors influencing consumers’ food waste can be categorized into three main groups, including societal (e.g., regulations, supply chain factors), personal factors (e.g., demographic, social norms, attitudes), and food-related practices (e.g., food planning and purchasing) (Stangherlin and de Barcellos 2018).

Regarding personal factors, studies have explored consumers’ subjective evaluations of food attributes and food values across food sources or acquisition channels. While food from local farmers is appreciated by consumers for many aspects (Cecchini et al. 2020; Feldmann and Hamm 2015), food sourced from supermarkets is associated with both negative and positive perceptions of its attributes (Brunori et al. 2016; van der Lans et al. 2014; Zhong et al. 2020). Leftovers, though being seen as a responsible way to reduce food waste (Kirmani et al. 2023), are often disregarded due to perceived quality deterioration, resulting in the disposal (Andrews et al. 2018; Aleshaiwi et al. 2021). The evidence above suggests that food sources are associated with perceived food attributes and food values. Coupled with findings that perceived food attributes and values guide food waste decisions (Aleshaiwi et al. 2021; Szymkowiak et al. 2022), this points to the plausible link between food sources and food waste behaviour. Nonetheless, this connection remains under-examined in the literature.

In this context, this study aims to investigate the influence of food sources on urban consumers’ food waste behaviour in Vietnam. Specifically, it compares food waste management practices across food sources and examines the impact of food sources on consumers’ decisions to save food. This study has four contributions. To our knowledge, it is the first attempt to examine the influence of food sources on food disposal decisions. Second, by using experimental and statistical methods, we were able to quantify the causal relationship between food sources and food waste behaviour. Third, this study addresses a documented gap in Southeast Asian research on food waste behaviour (Diana et al. 2024). Lastly, our research findings can be used as input for food waste reduction interventions, including consumer communication programs in Vietnam.

Vietnam offers a compelling context for this study. Food safety remains a dominant concern in Vietnam, as many prioritize assurances about chemical residues and hygiene when choosing vegetables and meat (Ha et al. 2019; Ngo et al. 2020; Nguyen-Viet et al. 2017). While price remains important for lower-income households (Trinh et al. 2023), consumers increasingly value safety, nutrition (Bell et al. 2021), and freshness (Dang et al. 2019). Trust in producers, brands, and known sources enhances willingness to pay for certified or local foods (Ngo et al. 2020). Different food distribution channels offer different food values to meet various consumers’ demands. For example, consumers who prioritize freshness or have lower incomes typically shop at traditional markets, whereas supermarkets offering safety-certified products primarily serve higher-income shoppers (Wertheim-Heck et al. 2019).

## Conceptual framework

In this study, we empirically test the effect of food sources on food waste behaviour. The hypothesized effect of food sources is informed by empirical evidence on the links (1) between food sources and perceived food values and (2) the link between perceived food values and food waste behaviour, as discussed below.

Previous studies have examined how food sources differ in perceived food values. Food purchased from local farmers or farmer markets, in consumers’ eyes, is associated with freshness, superior taste, and seasonality (Feldmann and Hamm 2015). Beyond these functional values, this local purchase is believed to promote a closer connection between end-consumers and local farmers (Benedek et al. 2018), supporting local communities and sustainable production (Cecchini et al. 2020). In contrast, food from supermarkets is often related to mass production and less freshness (Zhong et al. 2020). Nevertheless, they are characterized by convenience, low prices, wide variety (Brunori et al. 2016; van der Lans et al. 2014), and food safety assurances via labelling and quality standards (Trinh et al. 2020). Leftovers are often devalued by consumers due to their unappealing appearance, loss of taste (Aleshaiwi et al. 2021), and food safety concerns (Gjerris & Gaiani 2013; Andrews et al. 2018), making them undesirable or unwanted (Andrews et al. 2018; Aleshaiwi et al. 2021). However, gifted party leftovers might offer additional value. Food from social events is often believed as more authentic, homemade, or traditional, especially when shared among familiar groups (Hamburg et al. 2014; Hsieh et al. 2021). In addition, sharing leftovers may reinforce social bonds and hospitality norms, particularly in collectivist cultures (Porpino 2016) like Vietnam. In general, the above studies suggest a link between food sources and perceived food values.

The link between perceived food values and food waste behaviour has been explored by different domains. Findings from sociological and anthropological studies suggest that consumers are rational in food disposal decisions (Aleshaiwi and Harries 2021; Evans 2012; Farr-Wharton et al. 2014). According to Farr-Wharton et al. (2014), the values consumers place on a food product determine the willingness to rescue it. There is a common belief that good food cannot be thrown away (Evans 2012) and, therefore, food is wasted when it is perceived to have no value to individuals (Aleshaiwi and Harries 2021). The economics approach also assumes that consumers are rational decision makers (see Ang et al. 2021; Lusk and Ellison 2020), but offers a slightly different view. Drawing upon the maximum utility framework, it is argued that food is wasted when the utility of wasting exceeds the utility of saving. Utility, a related term of perceived values (Zeithaml 1988), is modelled as the difference between benefits and costs, which can be expressed in monetary and non-monetary terms (Ang et al. 2021). For example, the benefits of saving expired food are saving money (Ribbers et al. 2023) and positive emotions gained from “doing the right thing” (Graham-Rowe et al. 2015), while the perceived costs of saving might include food safety concerns (Nikolaus et al. 2018), potential health expense, and anticipated quality loss (Tsiros et al. 2005). Though the relationship between perceived values and food waste behaviour can be mapped by different approaches, a common ground among them is that the perceived values, rather than the actual values of food, are associated with food waste behaviour.

**Fig. 1:**
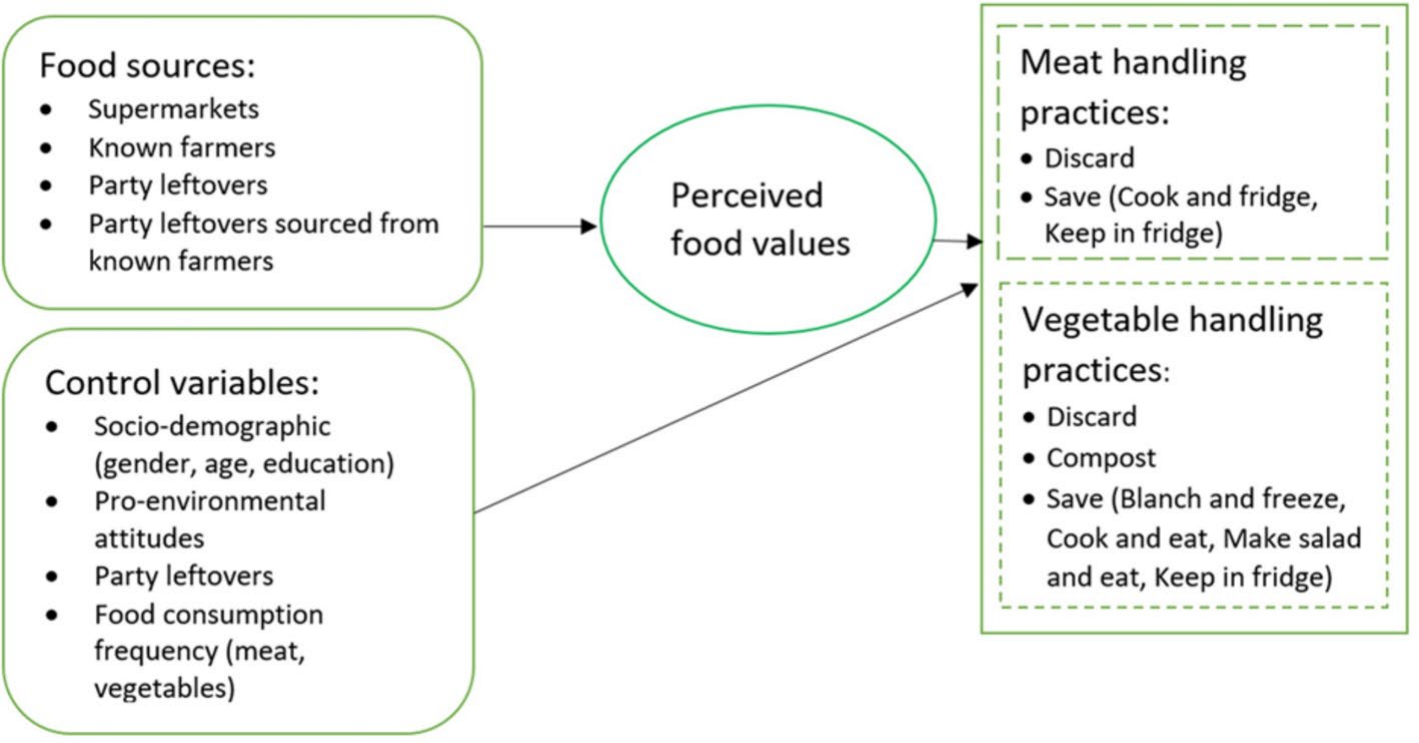
Conceptual framework

Figure 1 presents the theoretical framework of this study. Based on the above literature, we hypothesize that food sources influence food waste behaviour. Perceived food value is a potential pathway that connects the two. However, testing this pathway is not the focus of our empirical analysis.

Socio-demographic characteristics, pro-environmental attitudes, and the consumption frequency of the food are control variables. Younger consumers tend to waste more food than older individuals (Grasso et al. 2019). Food is wasted less among women, as compared to men (Grasso et al. 2019), possibly due to their greater involvement in food-related household tasks (Parizeau et al. 2015). Moroșan et al. (2024) found that education levels were positively associated with food waste frequency while Grasso et al. (2019) did not find the direct effect of education on consumer food waste behaviour.

Consumers with strong environmental concern are more likely to adopt waste-reducing practices, such as meal planning, freezing leftovers, and composting (Aktas et al. 2018; Visschers et al. 2016). However, the relationship between environmental concern and actual food waste behaviour is not always straightforward (Stancu et al. 2016). The frequency with which a food is consumed is likely to influence the decision to save or discard the food. Foods that are consumed regularly tend to be more familiar to consumers, which increases their confidence in the food’s safety, quality, and usability. This thereby strengthens the motivation to retain and use it.

In this study, we consider two hypothetical products, including vegetables and meat that are forgotten, expired, but still edible. Hereafter, we use ‘expired food’ to denote food items with these characteristics. We focus on four food sources for each product: food bought from supermarkets, food sourced from known farmers, gifted party leftovers, and gifted party leftovers with the food purchased from known farmers. Handling practices were classified into two main groups (“discard” and “save”) for meat and three groups for vegetables (“discard”, “save”, and “compost”). Composting also creates food waste; however, it might provide additional value by contributing to the circular economy, as compared to discarding.

## Methods

### Data collection

We conducted an online consumer survey in Vietnam in November and December 2022. The online survey was feasible and effective since the Internet is widely used in the country (Ministry of Information Technology of Vietnam 2022). We targeted consumers in the urban areas, which are defined as a place with high population density where people mainly engage in non-agricultural activities; a political/administrative, economic, cultural, or specialized centre that drives socio-economic development at national or regional scale (Vietnam National Assembly 2024). Studies from countries with similar social and cultural backgrounds to Vietnam, like China, show that an average urban consumer wastes more food than a rural consumer (Liu et al. 2023), and total household food waste in the urban region is much higher than in the rural region (Cheng et al. 2023). Due to the absence of household postal address databases and the focus on urban respondents, a snowball sampling technique based on professional networks was used. The survey link was distributed through social media platforms (i.e. Zalo and Facebook) to respondents living in urban districts of three big cities, namely Hanoi, Hai Phong, and Ho Chi Minh City, which together represent 20% of Vietnam’s population (GSO 2022).

The questionnaire was administered using Netigate, an online survey platform. Prior to the official survey, a pilot test was conducted with 20 participants. Total replies received were 1027. After data cleaning, 616 usable replies were obtained, with cases that failed the instructional manipulation check and vegan cases being removed (for more information about the survey, see Ha et al. 2024).

### Experiment design

The survey includes an experiment with four scenarios designed for each hypothetical meat (pork) and vegetable product. They are essential components of daily meals for most Vietnamese households. Nevertheless, these food items are perishable and associated with concerns about food safety in Vietnam, especially the fears of pesticide residues (for vegetables) and growth hormone (for meat) (Ha et al. 2019).

Participants were randomly assigned to one of four scenarios for both meat and vegetables, which were described as expired and forgotten, but still smelled fresh. The meat appeared visually acceptable, while the vegetables showed signs of wilting. The four scenarios present four different food sources: from a supermarket (the first scenario), from a known farmer (the second scenario), party leftovers distributed to the guests (the third scenario), and party leftovers bought from a known farmer distributed to guests (the fourth scenario). Based on the origin, we named scenario 1 as Supermarket, scenario 2 as Known Farmer, scenario 3 as Party Leftovers, and scenario 4 as Party Leftovers-Known Farmer.

For expired meat, participants could choose one of three options, namely discarding, cooking and freezing for later consumption, or storing in the fridge. For expired vegetables, respondents were asked to select one of six handling methods, including discarding, composting, blanching and freezing, cooking, making a salad, or storing in the fridge (Fig. 1). For meat, the scenarios read as follows:

“It is Monday night, 9 pm, you just had dinner and discovered that you have forgotten a pack of defrosted pork shoulder in the fridge that you […**words specific for scenario**…]. The meat expired yesterday, but it still looks and smells good. What do you do now?

- Scenario 1-Supermarkets: […words specific for scenario…] = [bought last week at the supermarket]
- Scenario 2-Known Farmers: […words specific for scenario…] = [bought last week from a small farmer you know who raises free-range pigs]
- Scenario 3-Party Leftovers […words specific for scenario…] = [brought back from the last barbecue party for which the host had bought too much and distributed the leftovers.
- Scenario 4-Party Leftovers-Known Farmers: […words specific for scenario…] = [you brought back from the last barbecue party, for which the host had bought too much and distributed the leftovers. The meat comes from a small farmer you know who raises free-range pigs.]

For vegetables, the scenarios read as follows:

“It is Monday night, 9 pm, you just had dinner and discovered that you have forgotten a pack of leafy green vegetables in the fridge that you […**words specific for scenario**…]. The vegetables look a bit wilted but still smell good. What do you do now?

- Scenario 1- Supermarket: […words specific for scenario…] = [bought last week in a supermarket]
- Scenario 2- Known Farmer: […words specific for scenario…] = [bought last week at the farmer market stall you regularly visit]
- Scenario 3- Party Leftovers: […words specific for scenario…] = [brought back from the last party for which the host had bought too much and distributed the leftovers].
- Scenario 4-Party Leftovers-Known Farmer: […words specific for scenario…] = [brought back from the last party for which the host had bought too much and distributed the leftovers. The vegetables were bought last week at the farmer's market stall you regularly visit.]

### Data analysis

Table 1 presents the socio-demographic characteristics of Vietnamese respondents across four scenarios. Sex distribution was relatively balanced in all scenarios. Respondents were young, with the average age ranging from 38.52 to 39.37 years old. Educational attainment was relatively high overall, with an average of 77% having a university degree, but highest in scenario 2 (around 84%) and lowest in scenario 4 (68%). 38% of the whole sample had income between 18 and 32 million VND per month.

**Table 1:** Socio-demographic characteristics of respondents across the scenarios

Variables	Variable description	Scenario (SC)			
		SC1 (*n* = 154)	SC2 (*n* = 153)	SC3 (*n* = 157)	SC4 (*n* = 152)
Sex (%)	Male	47.1	40.8	38.7	37.5
	Female	52.9	59.2	61.3	62.5
Age	Years old	38.9	39.4	38.5	39.1
Education (%)	No degree	0.7	0	0	0.7
	Primary school	0.7	0	0	0.7
	Secondary school	1.3	3.3	0.6	9.9
	Vocational education	5.2	3.3	3.8	1.3
	High school	3.9	3.9	6.4	9.9
	Higher technical	11.0	5.9	9.6	9.2
	University	77.3	83.7	79.6	68.4
Household income (%)	< 5 mil. VND*	2.6	0.7	1.9	4.0
	5–10 mil. VND	5.2	3.9	6.4	12.5
	10–18 mil. VND	20.1	26.8	26.8	21.1
	18–32 mil. VND	45.5	33.3	38.9	32.2
	32–52 mil. VND	14.9	22.9	17.8	19.1
	52–80 mil. VND	7.1	9.2	5.7	8.6
	> 80 mil. VND	4.6	3.3	2.6	2.6

*VND denotes Vietnam Dong. 1 USD = 23,600 VND, July 2023

Pearson's chi-squared test was used to determine whether there is a statistically significant difference between the expected frequencies and the observed frequencies in one or more categories of a contingency table. As a nonparametric test, Pearson Chi-square test is a widely used statistical method for testing hypotheses involving nominal variables (McHugh 2013). However, this test does not allow for testing the difference in specific food waste handling methods. Thus, to complement the Pearson Chi-square test, we used the two-sample proportion test, which is a parametric test for the difference of two proportions for independent samples. Using a two-sample proportion test, we examined whether the percentage of respondents selecting a handling method was equal within different pairs of scenarios. For every pair, the scenario with a lower proportion of respondents choosing to discard food can be considered more effective in reducing food waste. Analyses were performed using STATA 17.0.

To reveal the effect of food sources on the decision whether to save expired meat and vegetables, we applied a seemingly unrelated probit. Generally, a seemingly unrelated probit is considered a joint model for two correlated binary outcomes (Katchova 2013; Torres et al. 2016) and shares some of the independent variables. In this study, the application of a bivariate probit model is appropriate due to a potential correlation between the two outcomes: the likelihood to save vegetables and the likelihood to save meat. Food waste handling behaviour is habitual (Russel et al. 2017) if an individual tends to waste a food item, he/she is likely to waste other food products as well. The decision to save the two food products has two binary outcomes (= 0 if discarding or composting, = 1 if saving), which are specified by the two structural models as follows:

1 $$Y_{1}^{*} = \propto_{1} X_{1} + u_{1} \quad Y_{1} = \left\{ {\begin{array}{*{20}l} 1 \hfill & {{\text{ if}}\;Y_{1}^{*}> 0} \hfill \\ 0 \hfill & { {\text{if}}\;Y_{1}^{*} \le 0} \hfill \\ \end{array} } \right.$$

2 $$Y_{2}^{*} = \propto_{2} X_{2} + u_{2} \quad Y_{2} = \left\{ {\begin{array}{*{20}l} 1 \hfill & {{\text{if}}\;Y_{2}^{*}> 0} \hfill \\ 0 \hfill & { {\text{if}}\;Y_{2}^{*} \le 0} \hfill \\ \end{array} } \right.$$

where *Y*_1_* and *Y*_2_* represent the decisions to save expired meat and vegetables, respectively. *Y*_1_* and *Y*_2_* are unobserved latent variables, and their corresponding observed variables are *Y*_1_ and *Y*_2_. *X*_1_ and *X*_2_ are the vectors of corresponding covariates, while *α*_1_ and *α*_2_ are vectors of coefficients to be estimated. *X*_1_ includes scenarios (also food sources), age, sex, education, household income, meat consumption frequency, and pro-environmental attitudes. *X*_2_ covers the same set of variables as *X*_1_ but includes vegetable consumption frequency rather than meat consumption frequency. It is worth noting that the variable “scenario” is a categorical variable since each respondent was assigned to only one scenario (from scenario 1 to 4) and the values of these scenarios (1, 2, 3, 4) have no meaningful sequential order. Then we test if rho, which measures the correlation of the two error terms (*u*_1_, *u*_2_), significantly differs from zero, using the Wald test. The small p value of the test (*p* < 0.05) suggests the coefficient for rho statistically significantly differs from zero, and thus the bivariate probit model is appropriate. The parameters of Eqs. (1) and (2) were estimated in the command *biprobit* in STATA 17.0.

Table 2 presents the descriptive statistics and definitions of the variables included in the bivariate probit models. “Save_meat” and “Save_vegetables” are the two dependent variables. “Save_meat” was coded as 1 if respondents selected at least one of the two options: cooking and freezing, or storing in the fridge. “Save_vegetables” was coded as 1 if one of the following options was chosen: blanching and freezing, cooking, making a salad, or storing in the fridge. The remaining ones are independent variables. In terms of socio-demographic characteristics of the sample (*N* = 616), the average age was 39, and females accounted for 59% of the sample. Education level was relatively high (see Table 1), and this result is similar to previous studies conducted in urban areas of Vietnam (Ngo et al. 2020). In addition, the mean of pro-environmental attitudes, which assesses the importance of sustainable food production methods, was 5.1 based on a Likert scale from 1 (not important at all) to 6 (very important). On average, meat and vegetable were consumed 8.37 times and 12.89 times per week, respectively (see Table 2).

**Table 2:** Description of variables used in the bivariate probit models

Variables	Description	Mean	SD
Save_meat (*Y*1)	= 1 if save the meat (cooking and freezing, storing in the fridge) = 0 if discard	0.340	0.474
Save_vegetable (*Y*2)	= 1 if save the vegetables (blanching and freezing, cooking, making a salad, or storing in the fridge) = 0 if discard or compost	0.644	0.480
Scenario_meat	1: Reference scenario (meat purchased from a supermarket) 2: Free-range meat purchased from a known small-scale farmer 3: Party leftovers from a party host 4: Party leftovers, free-range meat purchased from a known small-scale farmer by a party host	2.505	1.118
Scenario_vegetable	1: Reference scenario (vegetables purchased from a supermarket) 2: Vegetables purchased from a known farmer's market stall 3: Party leftovers from a party host 4: Party leftovers, vegetables purchased from a known farmer's market stall by a party host	2.498	1.117
Age	Age of respondents (years old)	38.99	11.21
Sex	sex of respondents 1: female 0: male	0.59	0.49
edu	Education levels of respondents 1 (No degree), 2 (primary), 3 (secondary), 4 (vocational), 5 (high school). 6 (higher technical), 7 (university)	6.50	1.09
Pro-environmental attitudes	“Is it important that the food I eat on a typical day…*”:		
	Is produced in an environmentally friendly way	5.10	0.85
	Is prepared in an environmentally friendly way	5.11	0.92
	Is produced without disturbing the balance of nature	4.98	0.99
Meat consumption frequency	number of times per week that respondents eat meat (times)	8.37	4.87
Vegetable consumption frequency	number of times per week that respondents eat vegetables (times)	12.89	5.02

SD denotes standard deviation. * Items are adapted from Verain et al. (2021)

## Results and discussion

### Food waste management outcomes for meat and vegetables

**Fig. 2:**
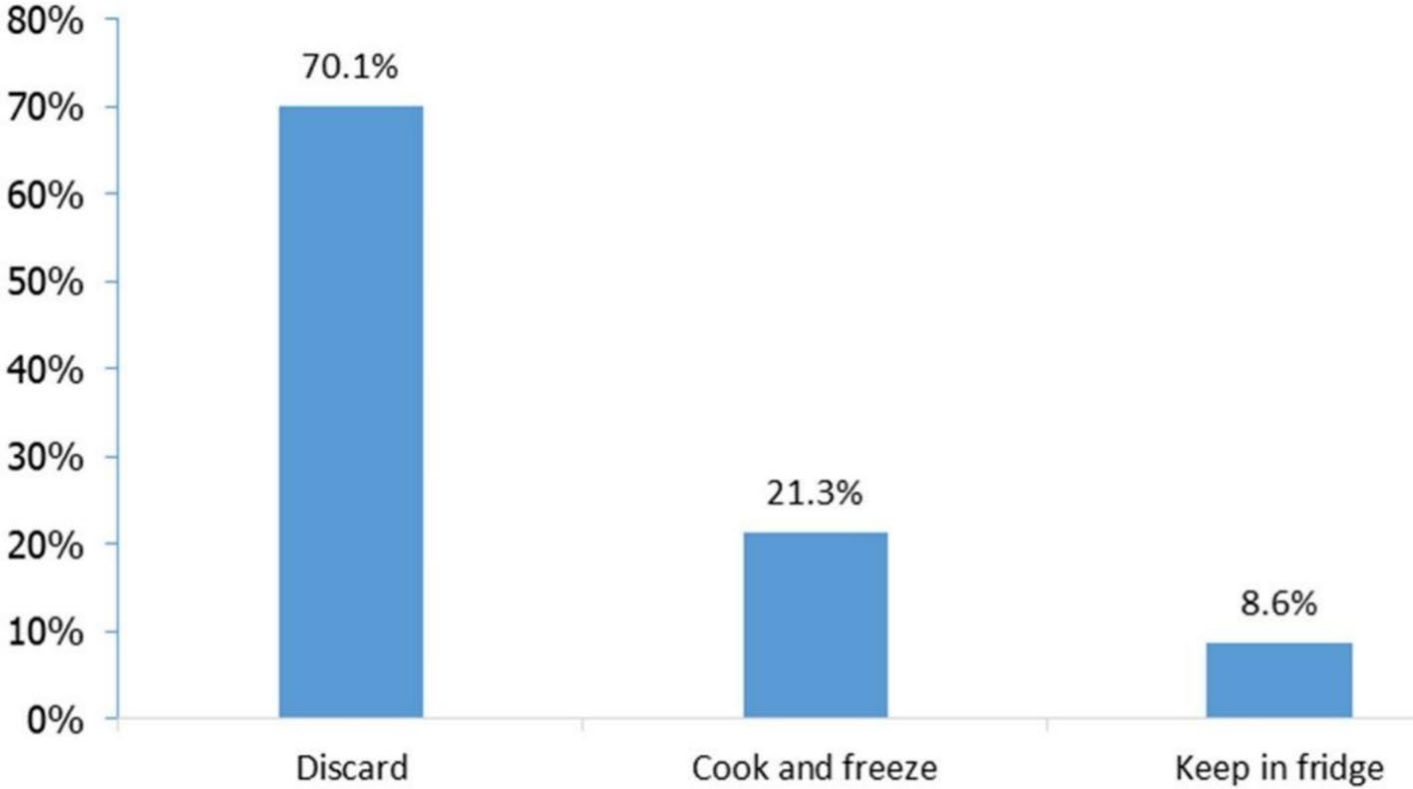
Percentage of respondents selecting handling methods for expired meat

**Fig. 3:**
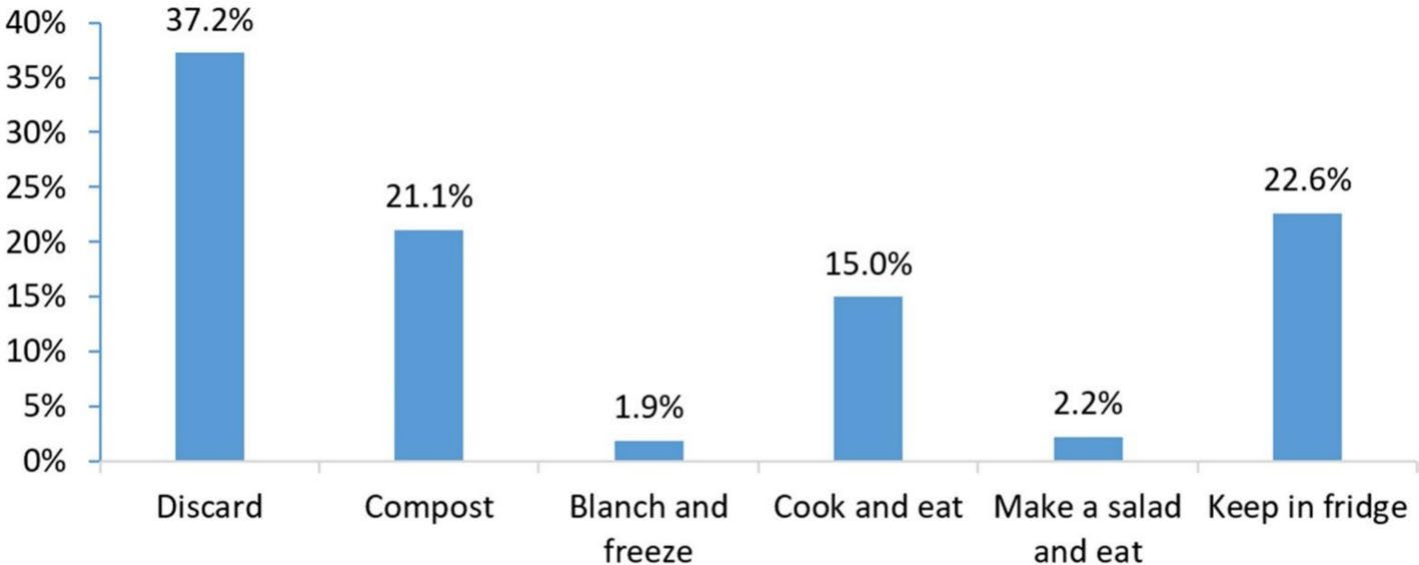
Percentage of respondents selecting handling methods for expired vegetables

Figures 2 and 3 present the aggregated percentage of respondents choosing food waste handling practices in all scenarios for meat and vegetables, respectively. The most commonly chosen practice for both meat and vegetables was discarding, with discard frequency being 70% for meat and 37% for vegetables. These high discard rates indicate the prevalence of food-wasting practices in Vietnam, which is also reported in a Food Bank’s study that found 87% of households wasted at least two food plates weekly.

The high food discard frequency can be explained by consumers’ anxiety about food safety in Vietnam, particularly pesticide residues in vegetables and growth hormones in pork (Ha et al. 2019; Ngo et al. 2020; Vuong et al. 2024). In our experiment, vegetables and meat, which are perishable and expired, might also cause food safety concerns among respondents. Since food perceived as abundant tends to be undervalued and discarded more readily (Aschemann-Witzel et al. 2015), it might also be the case that the high food availability in Vietnam might lead to low perceived food values, resulting in food wastage prevalence. Another possible explanation relates to the respondents’ characteristics, particularly their relatively high education levels and financial independence. Vietnam's daily vegetable supply per capita in 2022 is 501 g, far above the world average of 300 g (FAOSTAT and Food System Dashboard 2025). Pork availability has increased substantially in Vietnam in the past decade due to the drastic development of the domestic pig production and a strong growth in meat imports (Hansen 2018).

While meat is often more expensive than vegetables, a higher proportion of respondents chose to discard meat rather than vegetables. This may come from the higher food safety risk perceived for meat products compared to plant-originated products like vegetables (Djekic et al. 2022). Vietnamese consumers expressed great concern about the hygiene, biological contamination, and chemical hazards of meat (Ngo et al., 2021; Nguyen-Viet et al. 2019). Studies outside Vietnam also found the positive association between health concerns and the amount of wasted perishable food such as meat, fish, and dairy (Visschers et al. 2016). Pork, in particular, is among the food groups that are the most vulnerable to microbial contamination (Yang et al. 2022).

### Handling practices for expired meat by scenarios

Table 3 presents the percentage of respondents selecting food waste management practices for meat and the p-values of statistical tests across scenarios. Discarding meat was the most prevalent choice across all scenarios, with the highest proportion (77%) observed in the third scenario (party leftover meat) and the lowest frequency observed in the second scenario (meat purchased from a regular small-scale farmer) (62%). The proportion of respondents who discarded meat in the second scenario was significantly lower than in the first scenario (meat purchased from supermarkets) (scenario 2 vs 1: *p* = 0.016). This means that free-range meat purchased from a small-scale farmer was less likely to be discarded compared to meat purchased from supermarkets. The proportion of consumers who declared discarding meat also significantly differs between the fourth scenario (party leftovers sourced from regular small-scale farmers) and the first scenario (meat purchased from supermarkets) (scenario 4 vs 1: *p* = 0.025). The results also revealed that, comparing both scenarios with meat from leftovers origin (scenarios 3 and 4), the leftovers sourced from known farmers are less likely to be discarded (scenario 3 vs 4: *p* = 0.011). To summarize, our results suggest that Vietnamese consumers tend to put more value on meat obtained from regular farmers.

**Table 3:** Percentage of respondents selecting handling methods for expired meat and p values by scenarios

Handling methods	Percentage of respondents selecting handling methods by scenarios				*P* value based on Pearson's chi-square and proportion test					
	Supermarket (1)	Regular farmer (2)	Party leftovers (3)	Party leftovers- Regular farmers (4)	(2) vs (1)	(3) versus (1)	(4) versus (1)	(3) versus (2)	(4) versus (2)	(4) versus (3)
	*n* = 154	*n* = 153	*n* = 157	*n* = 152	0.087	0.237	0.207	**0.007**	0.303	0.184
Discard	74	62	77	67	**0.016**	0.766	**0.025**	**0.007**	0.868	**0.011**
Cook and freeze	20	30	14	22	0.107	0.151	0.954	**0.003**	0.094	0.167
Keep in the fridge	6	8	9	11	0.363	0.363	0.095	0.989	0.438	0.438

Values in “bold” indicate statistically significant differences at 5% significance levels. P values in the first row (the same row as sample sizes) are those from Pearson chi-squares and p values in the remaining rows are those from the proportion test

For cooking and freezing meat, a significant difference was only found between the second and the third scenarios, with more consumers willing to cook the meat if it was purchased from free-range small-scale farmers, compared to the party’s leftovers. In all scenarios, only 6% to 11% of respondents chose to keep the meat in the fridge for later use; however, no significant differences were found among the scenarios for this handling practice.

Since discarding is the most important answer option to reflect food waste handling behaviour in the scenarios, we discuss the results regarding this answer option only. There are two reasons why the meat in the second scenario (meat bought from a known small-scale farmer who raised free-range pigs) was less likely to be discarded, compared to meat bought from a supermarket. First, buying meat from a known farmer implies direct trade and an interpersonal relationship between the consumer and the producer, enabling trust building. Given concerns about pork safety in Vietnam (Dang-Xuan et al. 2016), trust in sellers helps reduce food safety concerns (Ha et al. 2020). These make them value meat from a known farmer more, resulting in a lower discarding rate, as compared to meat from supermarkets, where the interpersonal relationship between end-consumers and farmers is absent.

Second, the information on free-range pigs raised by a smallholder farmer might trigger perception of utilitarian (food safety, nutrition, sustainability), and hedonic values (good taste). In free-range farming systems, animals are free to stay outdoors, exposed to sunlight, and have natural feed (Liang et al. 2022; Situmorang et al. 2022). Thus, interest in free-range products is high (Liang et al. 2022; Situmorang et al. 2022), and consumers believe that these products are superior to conventional alternatives in terms of food safety, nutrients, taste (Situmorang et al. 2022), and animal welfare (Varziri et al. 2024). Food that is considered ethically produced, or locally sourced, often holds greater intrinsic value to consumers, leading to more deliberate storage and consumption practices (Mondéjar-Jiménez et al. 2016). In addition to the intrinsic values, it is possible that the utilitarian and hedonic values of free-range meat also motivate food waste reduction.

A higher percentage of respondents chose to discard party leftover meat with supermarket origin, as compared to free-range meat from a known small-scale farmer, suggesting that party leftovers were less valued by respondents. Perhaps the perceived costs of using leftover meat outweigh the perceived benefits. Wasting food is considered immoral by a proportion of consumers (Misiak et al. 2020). Receiving leftovers from others might reduce consumers’ moral concerns about food wastage and help them save food budget. However, party leftovers can evoke respondents’ negative emotions. Aleshaiwi et al. (2021) pointed out that leftovers that have been touched by others are considered unclean and disgusting. In addition, their unappealing appearance and the loss of sensory properties make leftovers undesirable (Aleshaiwi et al. 2021). Given these characteristics, party leftover meat seems inferior to free-range meat bought from a known farmer, leading to its higher likelihood of being wasted.

The results show that respondents placed a higher value on party leftovers sourced from a regular smallholder farmer than on party leftover meat bought in a supermarket. As previously mentioned, the hedonic values of the party’s leftover meat might be negatively evaluated. However, when the leftover meat is sourced from a regular smallholder farmer who is engaged in a free-range animal production system, such perceived negative values would become less salient, being cancelled out by perceived positive values offered by free-range meat, reducing the likelihood of discarding.

### Handling practices for expired vegetables across scenarios

Table 4 shows the percentage of respondents selecting food waste management practices for expired vegetables and p values across scenarios. Notably, discarding was the dominant response for vegetables bought from supermarkets, with 53% of respondents choosing this option, which is statistically significantly higher than vegetables from farmer markets (36%, *p* < 0.001) and party leftovers (27%, *p* < 0.001). This indicates a higher level of food waste associated with supermarket purchases. In contrast, party leftovers were more likely to be kept in the fridge (29%) compared to those from supermarkets (16%, *p* = 0.009) and farmer markets (17%, *p* = 0.015). These findings suggest that food acquired from social events like parties and personal connections, such as from farmer markets, encourages preservation behaviours, whereas food purchased from supermarkets leads to more wasteful practices. Other responses (i.e. composting, cooking, or making salad) did not differ significantly across scenarios (*p* > 0.05). Since composting also contributes to food waste, but the response frequency for the composting option did not statistically significantly differ across scenarios, we only discuss the result regarding the discard option and when mentioning wasting, we refer to the discard option.

**Table 4:** Percentage of respondents selecting handling methods for vegetables and p values by scenarios

Handling methods	Percentage of respondents selecting handling methods by scenarios				*P* value based on Pearson chi-square and proportion test					
	Supermarket (1)	Regular farmer (2)	Party leftovers (3)	Party leftovers—Regular Farmers (4)	(2) vs (1)	(3) vs (1)	(4) vs (1)	(3) vs (2)	(4) vs (2)	(4) vs (3)
	*n* = 154	*n* = 153	*n* = 157	*n* = 152	**0.017**	**0.000**	**0.000**	**0.008**	0.107	0.365
Discard	53	36	27	33	**0.000**	**0.000**	**0.000**	0.126	0.487	0.405
Compost	17	27	20	20	0.070	0.573	0.636	0.208	0.179	0.929
Blanch and freeze	1	2	3	1	0.652	0.255	0.566	0.479	0.317	0.102
Cook and eat	10	16	18	18	0.220	0.068	0.086	0.546	0.617	0.918
Make a salad and eat	3	2	3	1	0.702	0.993	0.414	0.708	0.657	0.419
Keep in fridge	16	17	29	28	0.876	**0.009**	**0.016**	**0.015**	**0.024**	0.852

Values in “bold” indicate statistically significant differences at 5% and less than 5% significance levels. *P*-values in the first row (the same row with sample sizes) are those from Pearson chi-squares and *p*-values in the remaining rows are those from proportion test

Similar to expired meat, expired vegetables purchased from a regular farmer were less likely to be wasted than those purchased from a supermarket, suggesting that the former is valued higher than the latter. In Vietnam, consumers often view food from regular vendors/farmers as safe (Wertheim et al. 2015). It is worth noting that the safety of food from supermarkets is also acknowledged by certain consumer groups in Vietnam (Hansen 2022) due to supermarkets’ food safety assurance systems, like certifications, food labels (Wertheim et al. 2015). Nevertheless, the safety and quality of vegetables sourced from a regular farmer might be evaluated even higher than those from supermarkets due to a higher level of trust in regular vendors (Ha et al. 2020). Food consumption based on personal trust gains importance in Vietnam (Kurfürst et al. 2019). Despite the growing popularity of supermarkets, a proportion of Vietnamese consumers rely on personal relationships with regular vendors to ensure food safety (Figuié et al. 2019). In contrast, trust in supermarkets varies (Figuié et al. 2019) and has partly eroded due to scandals in the past (Ha et al. 2020). Given a higher level of trust in regular farmers, food sourced from them might have higher perceived utilitarian, hedonic, and ethical values, resulting in a lower frequency of being wasted.

While party leftover meat was the most likely to be discarded (Table 3), party leftover vegetables were the least likely to be wasted (Table 4). A possible reason for this discrepancy is that party leftover vegetables might not evoke much disgust and concerns about foodborne diseases like leftover meat, which is perceived to be more vulnerable to microbial contamination (Djekic et al. 2022). Moreover, the loss of sensory properties of party leftover vegetables might be perceived as less serious than that of party leftover meat. Leftover vegetables might not cause high concerns about food safety risks and the negative perception of sensory attributes compared to leftover meat.

Gifted party leftover vegetables that the host bought from a regular farmer (scenario 4) were discarded less frequently than vegetables respondents purchased from a supermarket (scenario 1). This might be attributed to respondents’ positive perception of the values of party leftover vegetables, together with the perceived superior attributes of vegetables from a regular farmer, as previously discussed. Interestingly, when the information on the purchasing channel is absent, gifted party vegetables were also less likely to be wasted than vegetables sourced from supermarkets by respondents. Again, this result suggests the importance of food acquired from social events or personal networks. Compared to food bought from supermarkets, food acquired from personal networks might be trusted and valued more by consumers.

### The effect of food sources on the decisions to save food

Table 5 presents the results of the bivariate probit model. Overall, the interdependence assumption of the model was satisfied, as evidenced by the significant correlation coefficient between the error terms of the two sub-models (1 and 2) (rho = 0.399, *p* = 0.000).

**Table 5:** The results of the bivariate probit model: the decision to save expired meat and vegetables

Variables	Model 1		Model 2	
	Decision to save meat		Decision to save vegetables	
	Coefficient	Standard errors	Coefficient	Standard errors
Supermarkets	Reference		Reference	
Known farmers	0.361**	0.153	0.272*	0.152
Party Leftovers	− 0.056	0.160	0.641***	0.152
Party leftovers—Known farmers	0.385**	0.156	0.570***	0.152
Age	0.001	0.005	0.003	0.005
Sex	0.184	0.112	0.107	0.107
Education	0.052	0.053	0.064	0.051
Pro-environmental attitudes	− 0.011	0.061	− 0.041	0.060
Meat consumption frequency	0.037***	0.011		
Vegetable consumption frequency			0.004	0.010
rho	0.373***	0.062		
constant	− 1.353	0.546	− 0.970*	0.527
Observations	616		616	
Wald chi2(16)	62.95			
Prob > chi2	0.000			
Log likelihood	− 738.453			
Wald test of rho = 0: chi2(1) = 29.648 Prob > chi2 = 0.000				

The second scenario (food originating from local farmers) and the fourth scenario (party leftovers that the host purchased from known farmers) show a positive and significant effect on both decisions to save expired meat and vegetables. Specifically, compared to the reference scenario (food purchased from a supermarket), respondents in the second and fourth scenarios were more likely to save expired meat and vegetables. Moreover, since these two scenarios are both related to local food sources, the result above suggests that consumers valued local sourcing more and therefore were more reluctant to waste the food from this source. Particularly, the significant and positive effect of the fourth scenario for meat (*β* = 0.385, *p* = 0.01) and vegetables (*β* = 0.570, *p* = 0.000) reinforces the importance of local food and buyer–seller personal relationship in food waste reduction. Meanwhile, the third scenario (food comes from party leftovers) was non-significant in the meat-saving decision but exhibited a strong positive influence (*β* = 0.641, *p* = 0.000) on the vegetable-saving decision, as compared to the reference scenario. This finding suggests that, compared to vegetables respondents purchased from supermarkets, vegetables distributed to them as party leftovers were more valuable. Respondents may perceive that vegetables from party leftovers are of higher quality and associated with higher social values (the personal connection between the host and guests) than vegetables they purchased from supermarkets. In general, the regression results regarding the food sources are consistent with the results of the proportion test, which shows a lower percentage of respondents selecting the “discard” option for meat and vegetables with local origin, as compared to those with supermarket origin.

Demographic variables, including age, sex, and education were non-significant in both models 1 and 2, suggesting that they are unimportant predictors of food-saving decisions. Pro-environmental attitude was not significant in both decisions (Table 5). A possible reason for this finding is that consumers in Vietnam were not aware of the environmental issues (Markoni et al. 2023). Instead, they cared more about food safety and freshness, especially in meat and vegetables (Bell et al. 2021; Dang et al. 2019; Ha et al. 2019). In other words, saving expired food in Vietnam may be guided more by practical concerns, such as perceived food safety, sensory quality, than by abstract environmental motivations.

In this study, meat consumption frequency was positively associated with the likelihood of saving meat (*β* = 0.034, *p* = 0.000), while vegetable consumption frequency did not influence consumers’ decision to save vegetables (Table 5). Such differences can be explained by the fact that meat is more expensive than vegetables in Vietnam, which can motivate consumers with higher meat attachment to save meat. High consumption frequency might represent a high level of familiarity with a food product, which subsequently motivates respondents to save the food and reduce food waste (Pandey et al. 2023). It is also possible that the more frequently a food is consumed, the higher the perceived value it offers, which in turn reduces the likelihood of wasting the food.

Despite significant contributions to food waste behaviour literature, this study has some limitations. Since the survey was undertaken in urban areas of Vietnam, the research findings are not applicable to the rural population. In addition, this study did not empirically test the association between food sources and perceived food values, between perceived food values and food waste behaviour. Finally, participants were asked to imagine situations in which they discovered expired meat or vegetables under different food-source conditions. These scenarios assume that respondents can realistically simulate their reactions and decisions. However, real food waste-handling behaviours are influenced by sensory cues, time pressure, and social context, which are difficult to capture in written descriptions (Canali et al. 2017; Quested et al. 2013). To improve realism, future research could combine scenario-based surveys with observation studies to understand how consumers actually manage expired food. Such mixed designs would help validate whether intentions expressed in hypothetical settings align with real-life behaviour.

## Conclusion and policy implications

This is the first study to investigate how food sources influence consumer food waste behaviour. An experiment with four scenarios, which represent different food sources, was designed to capture food waste handling outcomes for hypothetical vegetable and meat products that were described as forgotten and expired, but still edible.

Four important findings are highlighted by this study. First, locally sourced vegetables and meat, which involve a personal relationship between end-consumers and farmers, were less likely to be wasted than vegetables and meat sourced from supermarkets, suggesting that local food was valued more by consumers. Second, party leftover meat that the host bought from supermarkets was most frequently discarded, possibly due to its potential costs (such as meat safety concerns) outweighing its potential benefits in consumers’ disposal decisions. Third, for leftover meat and vegetables that were locally sourced, the discarding frequency was lower than that of supermarket origin, suggesting that the negative perception about food safety and sensory attributes of leftovers has been weakened by the positive perception of locally sourced food. Fourth, in meat-saving decisions, meat consumption frequency was positively associated with a higher likelihood of saving.

Since food sources influence food waste handling behaviour, this relationship should be taken into account in interventions to reduce individual food waste. More specifically, since local food that involves personal connections between farmers and consumers is highly valued and less likely to be wasted, public policies that promote direct purchase or short food supply chains are needed. Support for various forms of direct purchase relevant to Vietnam’s context, such as farmer markets, trade fairs, box schemes, and group purchase in urban areas (see Paciarotti et al., 2021) can be one of the solutions to reduce food waste and food budget among consumers while improving farmers’ income. The highest discard frequency of vegetables from supermarkets underscores the need for targeted communication strategies by retailers to enhance the perceived value of this product and rebuild consumer trust. Additionally, given the potential link between food sources, perceived food values, and wasteful behaviour, educational campaigns that connect food values with origins that are appreciated by consumers can nudge them towards mindful food consumption. The high frequency of discarding meat highlights the need for consumer education aimed at raising awareness of the environmental impacts of meat consumption and improving handling skills in meat-related food waste prevention.

This study opens a new door for research that explores the relationship among food sources, perceived food values, and food waste behaviour. To test the link between perceived food values and food waste behaviour, experimental studies might use framing techniques to elicit different perceived values for the same food item from the same source. Alternatively, an experiment with different food sources, followed by survey questions asking respondents to rate different dimensions of perceived food values from each source, would be promising to test the link between food sources, perceived values, and food waste behaviour.

## Data Availability

Data will be provided upon reasonable request.
